# Condensation of an Additive-Free Cell Extract to Mimic the Conditions of Live Cells

**DOI:** 10.1371/journal.pone.0054155

**Published:** 2013-01-10

**Authors:** Kei Fujiwara, Shin-ichiro M. Nomura

**Affiliations:** Department of Bioengineering and Robotics, Tohoku University, Sendai, Japan; New England BioLabs, United States of America

## Abstract

The cellular environment differs from that of reconstituted materials mainly because of the presence of highly condensed biomacromolecules. To mimic the environment and conditions in living cells, we developed a method to prepare additive-free, highly concentrated cell extracts. First, we verified the requirement for specific salts and buffers for functional cell-free translation extracts. The S30 fraction of *Escherichia coli* cell extracts without additives exhibited sufficient cell-free protein production. Next, we established a method to accumulate biological components by gradual evaporation by using a vacuum desiccator. Bovine serum albumin, green fluorescent protein, alkaline phosphatase, and a diluted reconstituted protein expression system were successfully condensed in their active forms using this method. The protein concentration of the prepared cell extract was elevated to 180 mg/mL, which was expected to contain approximately 260 mg/mL macromolecules, without the loss of cell-free protein expression activity. Such a condensed cell extract may be useful for investigating the differences between cells and reconstituted materials and may contribute to the development of methods to synthesize cells from cell extracts in the future.

## Introduction

To understand biochemical reactions in cells, researchers have reconstituted various biological systems using purified components. For example, several studies have reported the reconstitution of DNA replication and transcription systems [1], [2]. Recent progress in molecular methodologies has permitted the reconstitution of the translation system, which includes approximately 100 components [3], and the assembly of membrane proteins required for cell division using purified components [4]. Thus, it is thought that combining the results of these reconstitution studies may enable the reconstitution of living cells from biological materials in the future.

Complicated biochemical systems have been analyzed by developing artificial cell models. Approaches to construct artificial cells, especially using liposomes as a model biomembrane in protocells, are alternative methods for reconstituting living cells [5], [6]. Cellular components encapsulated in liposomes have been used to study the behaviors of major macromolecules, i.e., DNA, RNA, and protein [7], [8], [9], [10]. Moreover, both cytosolic and membrane proteins can be functionally expressed within liposomes [9], [11], [12], [13]. For example, in a previous study, we expressed pore-forming membrane proteins in liposomes in order to enable the transport of bioactive peptides and small chemicals from liposomes to living cells [14]. These results suggest that artificial cells can be used to mimic living cells.

Although many studies have focused on the creation of protocells, reconstitution of living cells from protocells seems difficult. First, reconstitution of all essential cellular systems is a challenging problem. Second, identifying appropriate buffer conditions that are suitable for preparing biological components is difficult because inadequate buffer conditions may inhibit several chemical reactions. Furthermore, increasing the concentration of macromolecules to that found in living cells (i.e., approximately 300 mg/mL) [15] is a complicated task. Chemical reactions show different behaviors under high and low concentrations of macromolecules; this is termed the molecular crowding effect [16], [17]. Moreover, there might be other unknown factors that influence the reconstitution of cells from materials. Hence, these problems need to be addressed in order to achieve cell reconstruction.

Whole-cell extracts have been used as raw materials in many biochemical studies because they contain most cellular components and retain many biochemical activities found in living cells. Therefore, refining cell extracts may be the first step toward reconstructing living cells. However, a typical cell extract used for cell-free protein expression are only around 20–30 mg/ml of protein concentration and contains exogenous chemicals as buffers. Condensation of the cell extract and elimination of the exogenous chemicals used for preparing the cell extract may facilitate our understanding of the differences between living cells and cell extracts.

In this study, we prepared an *Escherichia coli* S30 cell extract without using exogenous chemicals and condensed this extract by gradual evaporation. Thus, we obtained a functional and high-concentration cell extract containing macromolecules at a concentration very similar to that found in living cells.

## Methods

### Proteins

Green fluorescent protein (GFP) was overexpressed in *E. coli BL21* (*DE3*) harboring pET15-sfGFP, in which the N-terminal of superfolder GFP (sfGFP) was 6×-His-tagged, and was purified using Ni-NTA agarose (Qiagen, Valencia, CA). Bovine serum albumin (BSA) was an appendix of BCA Protein Assay Kit (Thermo Fisher Scientific, Rockford, IL). PAP and PURE systems were purchased from Biodynamics Laboratory (Tokyo, Japan) and BioComber (Tokyo, Japan), respectively.

### Preparation of Double-distilled Water (DDW)-S30

*E. coli BL21* (*DE3*) cells (1 L) at approximately 1.0 OD_600_ were cultured at 37°C in LB medium. To obtain cold-shocked DDW-S30, the temperature was lowered to 16°C at 0.3 OD_600_, and the cells were further cultivated for 3 h. The harvested cells were suspended with 20% sucrose-water and incubated on ice for 10 min. They were then washed with 4 volumes of DDW, suspended in DDW, and incubated on ice for 10 min. The treated cells were washed twice with DDW, harvested, and stored for 1 day at −80°C without liquid nitrogen treatment. The frozen cells were disrupted using an S4000 sonicator (MISONIX, Inc., Farmingdale, NY) at 20 W for 15 min on ice. In this study, we used sonication to prepare cell extracts, unlike in typical studies on *E. coli* cell-free systems, because sonication enabled the preparation of high-concentration S30 (over 50 mg/ml) under our conditions. Next, 1 volume of DDW and 0.5–1 volumes of S30 buffer (10 mM Tris-acetate (pH 8.3), 60 mM potassium acetate, 14 mM magnesium acetate, and 1 mM DTT) were added to the cells to obtain DDW-S30 and Buf-S30, respectively. The disrupted cells were centrifuged at 30,000 × *g* for 1 h, and the soluble fractions obtained were the S30 fractions (DDW-S30, cold DDW-S30, or Buf-S30). Protein concentrations of S30 fractions were estimated using Bradford solution (Wako, Osaka, Japan) and BSA as a standard. The chemical concentration of S30 was estimated from the osmotic pressure by using a Vapro 5520KCE osmometer (Wescor Inc.). The concentration of nucleic acids in S30 was estimated from the absorbance at 260 nm determined using a Biospec-nano (Shimadzu, Kyoto, Japan) after treatment with phenol/CHCl_3_ twice and ethanol precipitation.

### Cell-free Protein Expression

The final concentrations of components used in the reaction mixtures were usually 50 mM 4-(2-hydroxyethyl)-1-piperazine ethanesulfonic acid (HEPES)-KOH (pH 7.6), 2 mM amino acids, 90 mM potassium glutamate, 15 mM magnesium acetate, 50 mM creatine phosphate, 200 µg/mL creatine kinase (Oriental Yeast), 1 mM nucleoside triphosphates (rNTPs) (Wako), 20 nM plasmid DNA, and 11 mg/mL S30 fraction. When indicated, several chemicals were omitted from the mixtures, and the final concentration of the S30 fraction was varied. The reaction mixtures were incubated at 37°C for 3.5 h. In the case of cold-shock expression, the reaction mixtures were incubated at 18°C for 16 h. The reaction was stopped using SDS-loading buffer (for sfGFP) or by placing on ice (for luciferase). For sfGFP and luciferase expression, pTD-sfGFP (a gift from Prof. Miyazaki, AIST, Japan) and pCold-Luc (a gift from Dr. Kato, Hyogo Pref Univ., Japan), respectively, were used (Fig. S1). DNAs were prepared using Qiagen mini-prep or maxi-prep kits. GFP expression levels were quantified by sodium dodecyl sulfate-polyacrylamide gel electrophoresis (SDS-PAGE) using unboiled samples. Fluorescent images were captured using a Molecular Imager FX Pro machine (Bio-Rad, Hercules, CA). Luciferase expression levels were quantified by measuring the luminescence intensity of the luciferase reaction mixture (500 µM ATP; 450 µM d-luciferin; 6.5 mM DTT; 13 µM coenzyme A; 4 mM magnesium acetate; 20 mM Tris-HCl, pH 8.0; and 1∶20 S30 reaction mixture) using a GelDoc XRS machine (Bio-Rad). The concentrations of sfGFP and firefly luciferase produced were estimated by comparing the obtained luminescence intensities with purified GFP or firefly luciferase. Relative amounts of each sample were quantified by using ImageJ software.

### Gradual Evaporation

Samples in 1.5-mL tubes (WATSON, Kobe, Japan) were incubated at room temperature in a vacuum desiccator (AS ONE, Osaka, Japan) with the lid open. The pressure of the vacuum pump was set at 0.1 M Pa. Samples were collected from the desiccator at certain periods as indicated. The total volume was measured using well-calibrated micropipettes (Gilson, Middleton, WI). Phosphate-buffered saline (PBS) concentration was estimated from the osmotic pressure using a Vapro 5520KCE osmometer (Wescor Inc.). The concentrations of BSA and DDW-S30 were estimated by the Bradford method. Active GFP was estimated by measuring the fluorescence intensity in a Nanodrop ND-3000 (Thermofisher-Japan, Yokohama, Japan). PAP activity was measured by colorimetric analysis. For this experiment, *p*-nitrophenyl phosphate (Wako) was used as the substrate, and the product levels were periodically estimated from absorbance at 405 nm using a Biospec-nano (Shimadzu). The condensation rates of volume or content used this study were the average of 3 independent samples.

## Results

### Preparing S30 Cell Lysates Using DDW

**Figure 1:**
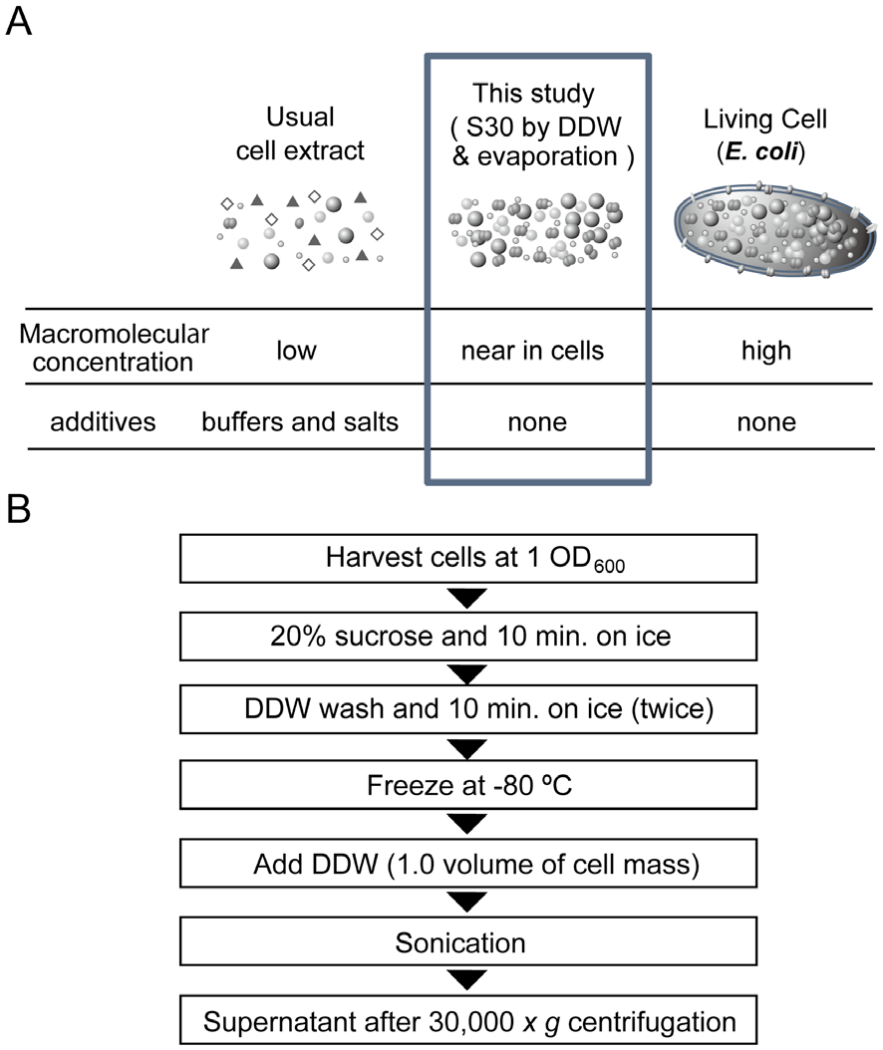
Aim and procedure for preparing DDW-S30. A: Scope of the present study. Preparation of *Escherichia coli* S30 with DDW and gradual evaporation enabled us to obtain an additive-free, high-concentration cell extract that showed similar macromolecular concentrations to living cells. B: Procedure for preparing DDW-S30. See details in the Methods section.

Buffers and salts are considered essential components required for the preparation of functional cell lysates, especially those for cell-free translation. The requirement for these chemicals was tested by preparing S30 cell lysates without these chemicals (Fig. 1). Late log phase *E. coli* cells grown in rich medium were harvested. The harvested *E. coli* cells were dissolved with 20% sucrose solution and washed with DDW. This treatment, known as cold-shock treatment, has been used to obtain periplasmic proteins during supernatant collection [18]. Thus, the cells remaining after the removal of DDW for washing were expected to contain fewer periplasmic proteins. Furthermore, this procedure enabled the replacement of buffer with DDW. Next, the cells were frozen at −80°C for 1 day and then sequentially dissolved in DDW. Subsequently, the cells were disrupted using sonication, and the DDW-S30 fraction was obtained from the disrupted cell lysates. This fraction typically contained around 50 mg/mL proteins, 25 mg/mL nucleic acids, and 70 mM chemicals.

### The DDW-S30 Extract Produced Functional Proteins

**Figure 2:**
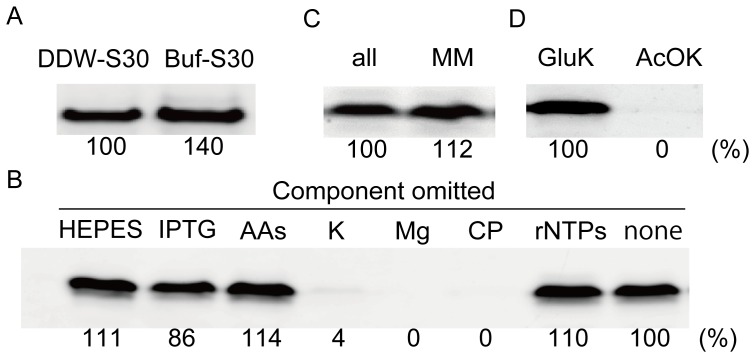
Cell-free protein expression by DDW-S30. The figure shows the GFP fluorescence for SDS-PAGE performed with unboiled samples. *A:* Cell-free GFP expression in S30 that was extracted in DDW and S30 with buffer addition. DDW-S30: S30 fraction extracted in DDW. Buf-S30: S30 fraction extracted using S30 buffer. *B:* Chemicals omitted from S30 reaction mixtures: HEPES, HEPES-KOH; IPTG, isopropyl β-d-1-thiogalactopyranoside; AAs, amino acids; K, potassium glutamate; Mg, magnesium acetate; CP, creatine phosphate; and rNTPs, nucleotides. “None” means that all chemicals were used. *C:* cell-free expression of DDW-S30 with minimum supplements. MM: mixtures of potassium glutamate, magnesium acetate, energy recycling system, DNA, and S30. All: mixtures of all chemicals used in *B*, DNA, and S30. *D*: Source of amino acids during the cell-free expression of DDW-S30 with minimum supplements. GluK and AcOK indicate that potassium glutamate and potassium acetate were used as the potassium source, respectively. Relative levels of sfGFP produced in each sample are indicated below the bands. The 100% indicates that 3.2 µM of sfGFP was produced in the sample.

The biological activity of the DDW-S30 lysates was evaluated by inducing cell-free protein expression (coupling transcription with translation). The *sfGFP* gene [19], which is stable at 37°C under a *tac* promoter (a strong promoter transcribed by the endogenous RNA polymerases), was used as a reporter for cell-free protein expression. The expression levels of sfGFP were estimated by measuring the fluorescence intensity by SDS-PAGE performed with unboiled samples. As a control, S30 lysates containing buffer and salts (Buf-S30) were prepared; in these lysates, typical S30 buffer was used instead of DDW [20], [21]. The S30 fractions were mixed with reaction mixtures for cell-free expression and plasmid DNA encoding sfGFP and incubated at 37°C. After 4 h of incubation, the expression levels of sfGFP were similar between the DDW-S30 and Buf-S30 fractions (Fig. 2A), indicating that endogenous chemicals are dispensable for the preparation of S30 for cell-free protein expression.

To further confirm this result, the DDW-S30 fraction was prepared from cold-shocked *E. coli* cells. Cold-shock expression induces a cold-shock response in cells and increases expression yields [22]. Before harvesting, the cells were incubated for 3 h at 16°C, and the S30 fraction was obtained from cold-shocked cells (cold DDW-S30). The expression level of the firefly luciferase gene, under the control of the cold-shock promoter (*cspA* promoter), was 3-fold higher at 18°C than at 37°C (Fig. S2). These results indicated that RNA polymerase, sigma factor, translational factors (including ribosomes), and the CspA transcriptional regulator for cold shock were functional in DDW-S30.

### Essential Additives for Cell-free Protein Expression by DDW-S30

Although DDW-S30 does not contain exogenous chemicals, several chemicals needed to be added to the reaction mixtures to induce cell-free protein expression. The requirement for each chemical was investigated to minimize the number of additives (Fig. 2B). Interestingly, removal of HEPES buffer, amino acids, or rNTPs did not affect the expression levels of sfGFP. Protein production by DDW-S30 in the absence of HEPES buffer may have resulted from contamination of the buffers with creatine kinase and/or DNA. On the other hand, potassium salts, magnesium salts, and energy-supplying systems, such as creatine phosphate or creatine kinase, were indispensable. These 3 agents were sufficient for inducing protein expression in our system (Fig. 2C). Protein expression was reduced when potassium glutamate was replaced with potassium acetate (Fig. 2D), suggesting that the amino acids required for protein production are derived from potassium glutamate. Cold DDW-S30 also expressed luciferase without amino acid supplementation (Fig. S2). The dispensability of amino acids and rNTPs was not unexpected because a previous study reported that glutamine and serine were sufficient for cell-free protein expression using S30 fractions [21].

### Protein Condensation by Gradual Evaporation

Evaporation is a simple, powerful means to condense chemicals. Concomitant condensation of exogenous chemicals used as buffers in the preparation of biological materials could result in unexpected dysfunctions of biological components. However, since DDW-S30 lacked exogenous buffers, this problem did not arise in the current study.

**Figure 3:**
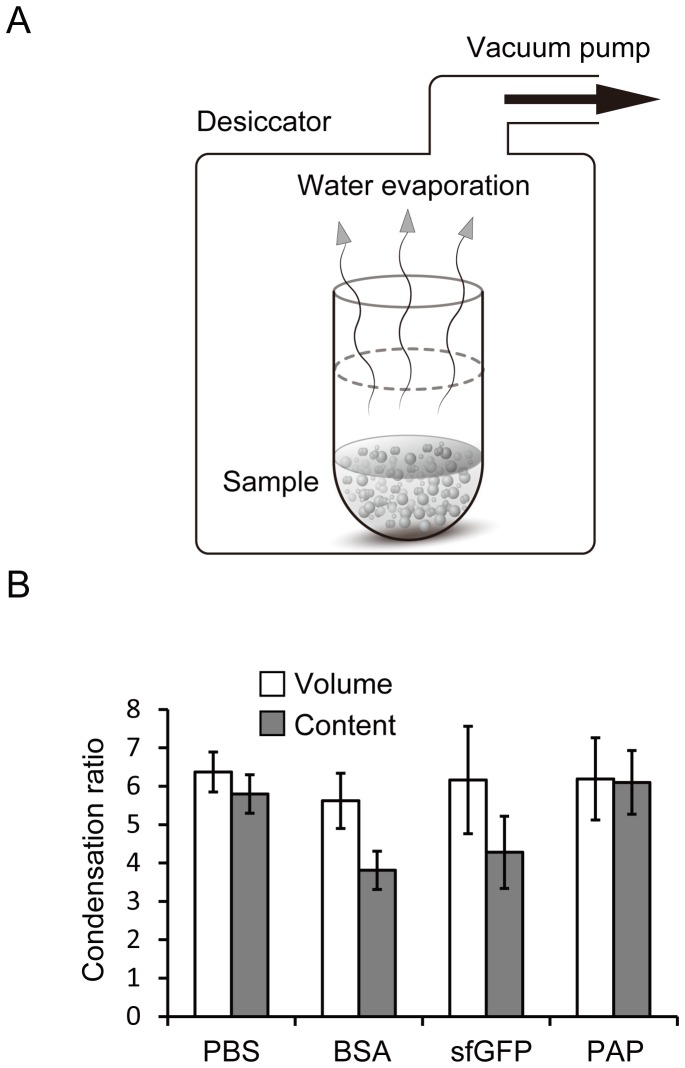
Residual ratios of the content after gradual evaporation. *A:* Schematic diagram of the condensation achieved by gradual evaporation. *B:* Condensation ratio of the total volume and content after gradual evaporation. Bars show the average condensation ratio (n = 3) of each sample. White bars: condensation ratio of volume, gray bars: condensation ratio of the content; these ratios were estimated from the osmotic pressure (PBS), protein concentration (BSA), fluorescent intensity (sfGFP), and activity (PAP). *PBS*, phosphate-buffered saline; *BSA*, bovine serum albumin; *sfGFP*, superfolder green fluorescent protein; and *PAP*, alkaline phosphate from a psychrophilic bacterium. Error bars indicate standard deviations of the condensation ratio after gradual evaporation.

Strong evaporation causes samples to dry and leads to denaturation of proteins. Hence, a gradual evaporation method was developed that avoided complete drying and could be applied to small amounts of biological components (Fig. 3A). Unlike traditional evaporation methods that require rotation of samples to speed up evaporation, in our method, samples were incubated in a vacuum desiccator at room temperature (around 25°C). Typically, the total volume of DDW decreased at a rate of 19 µL/h by gradual evaporation. Diluted PBS (0.2× PBS) evaporated slightly faster (rate, 21 µL/h) under the same conditions (Fig. S3). This method led to the evaporation of 10% of the components of PBS, retaining the rest.

Next, the evaporation of solutions containing BSA, GFP, and alkaline phosphatase (PAP) was investigated; condensation levels were estimated by protein concentration, fluorescence intensity, and activity (PAP) per theoretical level of condensation from the final volume, respectively (see Methods section; Fig. 3B and Table S1). BSA and GFP condensed at a similar rate (5.62-fold condensation for BSA, 6.16-fold condensation for GFP), and 70% of the components remained after a 3.5-h evaporation. After the 3.5-h evaporation, PAP was condensed to a 6.19-fold higher concentration, and 100% of the components were retained. The high condensation rate of PAP may have been due to the presence of detergent (Triton X100) in the buffer. Temperature conditions affected the evaporation speed. Evaporation at 28°C (3°C higher than the usual temperature) resulted in a 29.0-fold condensation of PAP. The degree of PAP condensation did not alter the condensation rates of its content (Fig. 3B).

### The Concentration of Components in the Condensed DDW-S30 was Similar to that in Living Cells

**Figure 4:**
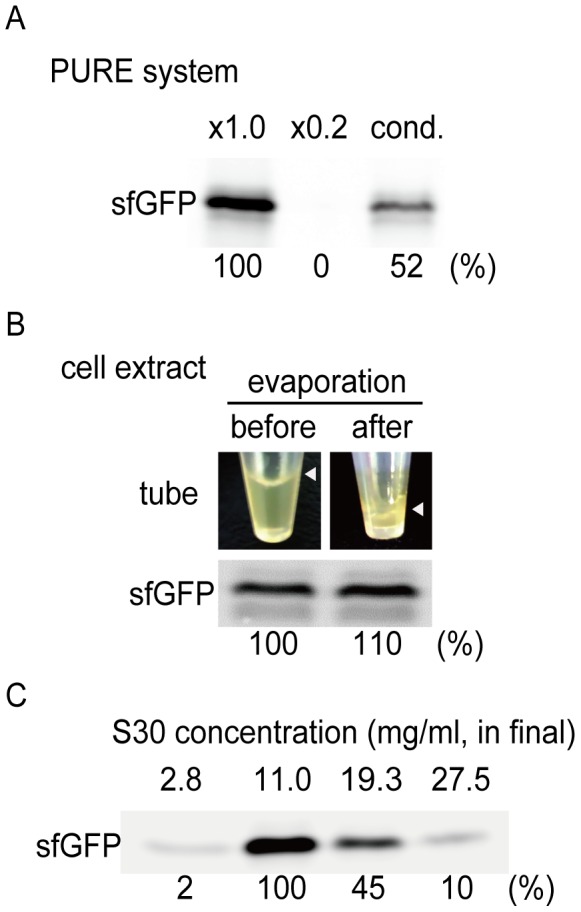
Condensation of complex systems by gradual evaporation. *A:* Condensation of the diluted PURE system. For this, 50 µL of sample was evaporated for 3.5 h at room temperature. GFP was expressed from DNA in 1.0× (not diluted PURE system), 0.2× (PURE system diluted 5-fold in DDW), and cond. samples. (condensed sample of the diluted PURE system from 0.2 fold to 1.0 fold concentration). *B:* Condensation of DDW-S30. The top panel shows DDW-S30 in the tube before and after 3.5 h evaporation. The bottom panel shows sfGFP expression from 11 mg/mL of DDW-S30 before and after the evaporation. Arrows indicate the upper surface of the solution. *C:* Dependence of cell-free expression on the final concentration of DDW-S30. *A* and *bottom panel of B:* sfGFP fluorescence on SDS-PAGE. Relative levels of sfGFP produced in each sample are indicated below the bands.

Next, we investigated the condensation of a reconstituted protein expression system (PURE system) containing more than 100 factors. The PURE system reaction mixture was diluted 4-fold with DDW (0.2× PURE system) and then concentrated using the gradual evaporation method. After evaporation, the volume of the concentrated mixture, which was more than 5-fold greater than the 0.2× PURE system mixture by volume, was adjusted to the volume of the 1× PURE system. The condensed sample showed 52% protein expression activity compared with that of the undiluted PURE system (Fig. 4A); this finding may have been attributable to the denaturation of proteins or loss of samples. Nonetheless, the protein expression efficiency of the concentrated PURE system was higher than that of the diluted system, indicating that the evaporation method can be applied to complicated biological mixtures.

DDW-S30 was also condensed using the gradual evaporation method. After evaporation for 3.5 h, the protein concentration of the cell extract reached 180 mg/mL (Fig. 4B). Cell-free protein expression levels of the condensed DDW-S30 were almost similar to those of the uncondensed DDW-S30 (Fig. 4B). As expected, the cell extract was more stable than the PURE system after condensation because the PURE system does not contain any factors associated with protein stability, such as chaperones [23]. In terms of nucleic acid concentration, our data demonstrated that the condensed DDW-S30 mixture contained RNA and DNA at 0.5-fold the amount of protein. Thus, we concluded that the concentrated DDW-S30 was expected to contain 260 mg/mL of macromolecules.

Generally, enzymatic activity increases in proportion to the concentrations of enzymes. Thus, the protein expression levels of concentrated DDW-S30 were expected to be higher. However, the protein expression efficiency of uncondensed DDW-S30 having a protein concentration of over 19 mg/mL decreased in proportion to the concentration of the cell extract (Fig. 4C).

## Discussion

Obtaining high-concentration functional cell extracts has, until now, been a significant limitation to the reconstitution of whole cells from cell extracts. In this study, functional cell extracts were prepared without exogenous chemicals (DDW-S30) and were concentrated using gradual evaporation. Like in living cells, metabolites may have acted as buffers in the presence of DDW, and molecular chaperones in the cell extract may have stabilized the proteins under evaporation conditions. Indeed, we were able to obtain a concentrated DDW-S30 extract having a macromolecular concentration that was almost the same as that in living cells. This finding can be applicable to studies on the molecular crowding effect, which causes a remarkable gap between in vitro assay systems and cells. Molecular crowding reagents such as polyethylene glycol (PEG), dextran, and Ficoll have been used to study the molecular crowding effect. However, these chemicals have somewhat different characteristics and have shown opposite effects in several cases [16], [24], [25].

A major concern regarding DDW-S30 was that when the protein concentration was more than 19 mg/mL, the efficiency of protein expression decreased (see Fig. 4C). Although several studies have reported that condensation of S30 increases the total protein expression levels [26], [27], the final concentration of S30 in those studies (10–15 mg/mL) was not as high as that used in this study (i.e., 19–28 mg/mL), where decreased efficiency of protein expression was observed. In living cells, the protein concentration is more than 200 mg/mL, and the protein expression system is functional, even under such high concentrations of macromolecules. Moreover, the expression of sfGFP in living cells was more than 10-fold higher than that in our cell-free protein expression system. Therefore, the expression efficiency of proteins in DDW-S30 may be improved at even higher concentrations (more than 19 mg/mL final concentration of S30) if the conditions available in living cells are mimicked. However, additional unknown factors that would help mimic living cells are also required to facilitate the reconstruction of living cells. Omics studies, such as metabolomics and proteomics, which were performed in our previous chaperone study [28], may contribute to the identification of such factors. Unlike in cell extracts, macromolecules inside living cells are distributed in a regulated fashion, with different local concentrations according to function. Such dynamic but ordered structural regulation may be disrupted by cell extraction. Nevertheless, we believe that investigation of differences and similarities between living cells and the concentrated S30 in this study is an important process that will facilitate our understanding of cells as complex biological systems.

A previous study reported that the macromolecular concentration in live *E. coli* cells in the log phase of growth is approximately 300 mg/mL and that protein and RNA account for about 70% and 30% of macromolecule content, respectively, indicating total RNA mass is approximately 0.43-fold that of protein mass [15]. About 95% of total RNA is accounted for by rRNA and tRNA; these RNA forms are essential for translation. Since DDW-S30 retained cell-free translational activity after evaporation, even in the absence of these RNAs, the condensed DDW-S30 was expected to have this level of RNAs. Indeed, in the current study, the condensed DDW-S30 contained nucleic acids (including RNA and DNA) at 0.5-fold the amount of protein.

Existence of functional membranes is an obvious distinction between living cells and cell extracts. Cells essentially have membranes with proteins, but cell extracts do not. Uptake and efflux of low-molecular-weight molecules are essential for efficient protein production, as has been shown during the continuous expression of a cell-free protein production system using dialysis in tubes or liposomes [12], [29]. In addition, a very recent study suggests that the lipid membrane itself might accelerate the rate of protein expression [30]. Hence, entrapping the concentrated DDW-S30 in a lipid membrane may be useful in the reconstitution of living cells.

## Supporting Information

Figure S1 **Regions of the plasmids used in this study.** RBS: ribosome binding sites (SD sequences), sfGFP: superfolder GFP gene, RIT: a Rho-independent terminator, and UTRs: untranslated regions of *cspA*, Accession numbers are AFM44944 for sfGFP and BAL46511 for firefly luciferase.
(TIF)

Figure S2 **Expression of luciferase under the cold-shock promoter by DDW-S30.** Usual: reaction was performed at 37°C for 3.5 h; Cold: reaction was performed at 18°C for 16 h (cold-shock condition); and AA-: the reactions mixtures did not contain amino acids under cold-shock condition. The relative activity was determined by measuring the chemical luminescence intensity of the reaction mixture without DNA as 0 and that of the reaction at 37°C as 1. One unit is equivalent to 0.04 µM of purified firefly luciferase.
(TIF)

Figure S3 **Decrease in the total volume by gradual evaporation.** Initial sample volumes were 100 µL. The blue line indicates the result of DDW, and the red line indicates the result of the diluted PBS.
(TIF)

Table S1 **Condensation factor of volume and content by gradual evaporation.**
(RTF)
