# Supplementary figures and images for: Condensation of an Additive-Free Cell Extract to Mimic the Conditions of Live Cells
Source: PLoS One. 2013 Jan 10;8(1):e54155. doi: 10.1371/journal.pone.0054155 (PMC3542322; doi:10.1371/journal.pone.0054155)

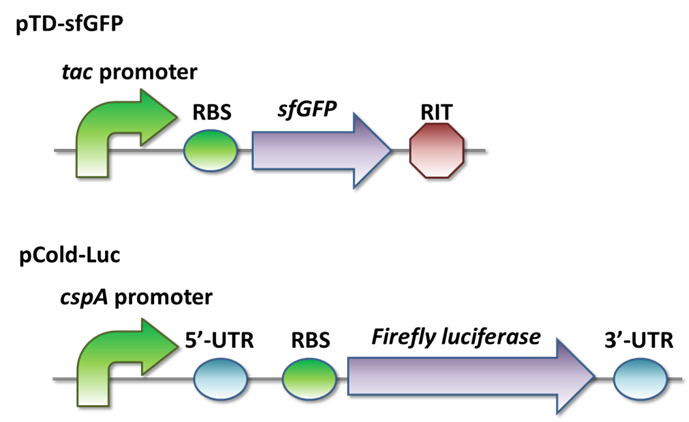

Supplement: Figure S1 — Regions of the plasmids used in this study. RBS: ribosome binding sites (SD sequences), sfGFP: superfolder GFP gene, RIT: a Rho-independent terminator, and UTRs: untranslated regions of cspA, Accession numbers are AFM44944 for sfGFP and BAL46511 for firefly luciferase. (TIF) [file pone.0054155.s001.tif]

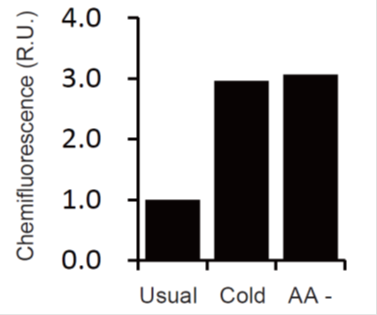

Supplement: Figure S2 — Expression of luciferase under the cold-shock promoter by DDW-S30. Usual: reaction was performed at 37°C for 3.5 h; Cold: reaction was performed at 18°C for 16 h (cold-shock condition); and AA-: the reactions mixtures did not contain amino acids under cold-shock condition. The relative activity was determined by measuring the chemical luminescence intensity of the reaction mixture without DNA as 0 and that of the reaction at 37°C as 1. One unit is equivalent to 0.04 µM of purified firefly luciferase. (TIF) [file pone.0054155.s002.tif]

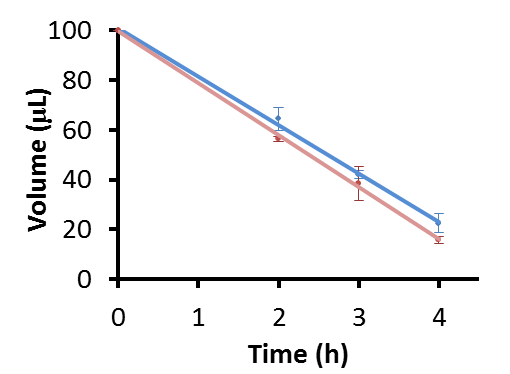

Supplement: Figure S3 — Decrease in the total volume by gradual evaporation. Initial sample volumes were 100 µL. The blue line indicates the result of DDW, and the red line indicates the result of the diluted PBS. (TIF) [file pone.0054155.s003.tif]
