# Supplementary material for: Condensation of an Additive-Free Cell Extract to Mimic the Conditions of Live Cells
Source: PLoS One. 2013 Jan 10;8(1):e54155. doi: 10.1371/journal.pone.0054155 (PMC3542322; doi:10.1371/journal.pone.0054155)
Supplement: Table S1 — Condensation factor of volume and content by gradual evaporation. (RTF) [file pone.0054155.s004.rtf]

Samples	Condensation factors
(Volume)	Condensation factors
(Content)	Evaporation time (h)	
DDW	4.55 ± 0.70	N.D.	4 	
0.2× PBS	6.37 ± 0.52	5.80 ± 0.50	4 	
BSA	5.62 ± 0.72	3.81 ± 0.50	3.5 	
sfGFP	6.16 ± 1.40	4.28 ± 0.94	3.5 	
PAP (25°C)	6.19 ± 1.07	6.10 ± 0.83	3.5 	
PAP (28°C)	29.0 ± 12.8	26.7 ± 8.9	3.5	
